# A Graph-Related High-Order Neural Network Architecture via Feature Aggregation Enhancement for Identification Application of Diseases and Pests

**DOI:** 10.1155/2022/4391491

**Published:** 2022-05-26

**Authors:** Jianlei Kong, Chengcai Yang, Yang Xiao, Sen Lin, Kai Ma, Qingzhen Zhu

**Affiliations:** ^1^School of Artificial Intelligence, Beijing Technology and Business University, Beijing 100048, China; ^2^Research Center of Intelligent Equipment Technology, Beijing Academy of Agriculture and Forestry Sciences, Beijing 100097, China; ^3^School of Engineering, Beijing Forestry University, Beijing 100086, China; ^4^School of Agricultural Equipment Engineering, Jiangsu University, Zhenjiang, Jiangsu 212013, China

## Abstract

Diseases and pests are essential threat factors that affect agricultural production, food security supply, and ecological plant diversity. However, the accurate recognition of various diseases and pests is still challenging for existing advanced information and intelligence technologies. Disease and pest recognition is typically a fine-grained visual classification problem, which is easy to confuse the traditional coarse-grained methods due to the external similarity between different categories and the significant differences among each subsample of the same category. Toward this end, this paper proposes an effective graph-related high-order network with feature aggregation enhancement (GHA-Net) to handle the fine-grained image recognition of plant pests and diseases. In our approach, an improved CSP-stage backbone network is first formed to offer massive channel-shuffled features in multiple granularities. Secondly, relying on the multilevel attention mechanism, the feature aggregation enhancement module is designed to exploit distinguishable fine-grained features representing different discriminating parts. Meanwhile, the graphic convolution module is constructed to analyse the graph-correlated representation of part-specific interrelationships by regularizing semantic features into the high-order tensor space. With the collaborative learning of three modules, our approach can grasp the robust contextual details of diseases and pests for better fine-grained identification. Extensive experiments on several public fine-grained disease and pest datasets demonstrate that the proposed GHA-Net achieves better performances in accuracy and efficiency surpassing several other existing models and is more suitable for fine-grained identification applications in complex scenes.

## 1. Introduction

Agriculture is an industry that integrates technology, economy, politics, and national security, supporting the entire history of human evolution, and is increasingly multifunctional with social development and progress. Agriculture is an essential source of basic living materials for human society, which provides the primary products and material basic conditions for developing the global economy and feeding humanity [1, 2]. Plant pests and diseases affect the overall functioning of the plant, and it can lead to slowed growth, reduced fruit yield, leaf drop, and many other diseases. The sources of plant diseases are multifaceted. Sometimes, the diseases are spread from one crop to another caused by some fungus or other bacteria. Moreover, some viruses hidden in the seeds are transferred from one place to another place, resulting in outbreaks of diseases [3]. Due to the complex generation and transmission mechanism, pests and diseases have become one of the most challenging questions faced by global agricultural production. According to statistics, the annual loss of plant yields due to diseases and pests accounts for more than 50% [4]. Therefore, the accurate identification of disease and pest species is of great significance to controlling plant diseases and pests as well as improving crop yield.

Identifying diseases and pest maintenance in traditional agricultural production relies on some agronomy experts with professional knowledge and experience through their naked eyes [5, 6]. This approach is usually a time-consuming, uneconomical, and repeated job when invasive species appear or the production farmlands are changed, which affects the subsequent production operations. In recent years, modern agriculture is striving to make full use of innovative systems and methods to achieve intelligent production and management, including Internet of Things (IoT), automated robots, big data cloud computing, and artificial intelligence modelling [7–11]. With the development of information and intelligent technologies, the automatic and accurate identification methods handling various diseases and pests have become one of the important issues in the modern agricultural production process, which attracts many agricultural experts and enterprises to carry out related research [2, 12–14]. An efficient and reasonable solution is using some machine learning-based methods to identify the species of plant pests and diseases [15–17]. On basis of enough image data collected by visible sensors or devices, those methods firstly apply some classical statical learning or machine learning methods to identify the varieties of plant pest and diseases. Those classical methods mainly rely heavily on complex statical analysis and designed feature engineering to gain a modest performance, which occupy massive computing power and human resources to cover time-consuming model training process, leading to a lack of recognition ability in complex practical applications.

With the development of information technology and intelligent approaches, deep learning neural networks have made significant improvement in solving many intelligent applications, including image classification, object detection, and video tracking, which provide a promising candidate for a generalizable approach for complex environment practices. At present, several deep learning models, including convolutional neural network (CNN), recurrent neural network (RNN), and generative adversarial networks (GAN), have been introduced into the visual recognition of plant's disease and pest species [18–20]. With multilayer structure and massive majorization modules, deep learning methods could automatically learn the multidimensional feature expressions from a large amount of raw data and mine more potential rules at an abstract perspective, which obtained better accuracy and stronger robustness over traditional technologies in identifying different plant pest and disease types.

Although many studies provide credible references to demonstrate the superiority of deep learning approaches, accurately classifying plant diseases and pests is still challenging in a natural scene. The main problem is that the practical identification process of different diseases and pests is a typical fine-grained visual classification (FGVC) problem [21]. As a research hotspot of artificial intelligence in recent years, FGVC is mainly to identify image samples belonging to multiple sublevel classes via retrieving objects under a meta-level category, which is more complicated than simple coarse-grained identification of existing deep learning methods [22–25]. There are many fine-grained difficulties that need to be addressed in identifying plant diseases and pests. On the one hand, the same meta-level category contains vast sublevel samples with viewpoints, illumination, positions, growth periods, and environmental factors, which are easy to confuse the model, resulting in incorrect identification. On the other hand, there is a certain similarity among different meta-level categories. Different genera or subspecies of the same biological subject often have a highly similar overall appearance except for several critical local parts. Thus, deep learning models based on coarse-grained representation learning are generally lacking the effective and accurate ability to handle the identification puzzle for plant disease and pest.

To overcome the above problems, this study proposes a novel graph-related high-order architecture with feature aggregation enhancement to establish a fine-grained deep learning neural network (GHA-Net), aiming to promote the rapid identification of plant pests and diseases in complex natural applications. There are two main innovations in this proposed method. Firstly, a feature aggregation module is designed to improve the fine-grained feature perception capability of the cross stage partial network stage (CSP-stage) backbone network, which filters out multiscale discriminating features and pays more attention to local parts. Secondly, the graphic convolution module is constructed to analyse the graph-correlated representation of part-specific interrelationships by regularizing semantic features into the high-order tensor space. With collaborative learning and global optimization, our approach can achieve better fine-grained identification performance for massive plant diseases and pests in terms of accuracy and robustness, which is more suitable for practical recognition applications.

The rest of the paper is organized as follows: Section 2 introduces the related research of computer vision and machine learning techniques for plant pest and disease identification. Then, the details of the proposed algorithm and experimental datasets are explained in Section 3.  Section 4 presents contrastive experimental results and performance evaluation. Finally, Section 5 concludes the whole work with future research prospects.

## 2. Related Work

Plants infected with diseases and pests usually exhibit visible marks or lesions on the leaves, stems, and fruits, which generally present unique visible patterns for intelligent diagnosis. Nowadays, lots of researchers apply machine learning and information processing techniques to identify different pest and disease species on controllable environmental conditions or public competition datasets. In this section, we review and summarize some relevant studies on machine learning technologies and coarse-grained deep learning methods for plant disease and pest classification, as well as some fine-grained recognition approaches, which is also a key issue of our work.

### 2.1. Machine Learning Technology for Plant Disease and Pest Identification

In order to guarantee the amount of training data for training complicated deep learning models, many studies have collected various public datasets of plant disease and pest categories. In the first stage of intelligent algorithm modelling, a sufficient amount of image data is necessary for feature extraction, including texture, shape, color, and motion-related attributes. Islam et al. [22] used a dataset of 300 potato leaves from the PlantVillage dataset [26] to design a classifier capable of identifying healthy leaves and leaves affected by late blight and early blight. Then, a multiclass support vector machine was used to classify leaf images based on ten colors and texture features. Qin et al. [27] proposed a solution to identify four leaf diseases affecting alfalfa grass by cropping the image to generate a subimage with one or more lesions. At the same time, the principal component analysis (PCA) method was used to reduce the dimension of *45* feature vectors, which offers the Naïve Bayes classifier to achieve good classification results. Similarly, Wu et al. [23] collected over *75,200* images covering *102* types of crop pests and framed more than 19,000 photos to handle visual classification and detection. Recently, some public competitions also provided public pest and disease datasets, such as AI Challenger 2018, which provided nearly 50,000 photos of plant leaves classified into 61 categories. Cassava Leaf Disease Classification competition provided a dataset of *21,367* labeled images of cassava, divided into four disease classes and health states [28]. These datasets often contain disease and pest infestations, providing a good foundation for intelligent algorithm modelling and optimization.

Meanwhile, several statistical learning and machine learning approaches have been widely introduced to the image identification of plant diseases and pests, including support vector machine (SVM), K-nearest neighbour (KNN), decision tree (DT), and artificial neural network (ANN). For example, Kumar et al. [29] designed an exponential spider-monkey algorithm to optimize an SVM-based classifier, which selects the high-dimensional essential features from the images of pests and diseases by eliminating unimportant features. Zhang et al. [30] proposed a hybrid clustering-based method for plant disease and pest identification. Through KNN clustering, the entire image is divided into several compact and approximately uniform superpixels, providing valuable clustering clues for guiding image segmentation, which effectively detects plant diseases and pests. Similarly, Khule et al [31] attempted to apply different machine learning methods for the classification of tomato diseases. Using image processing techniques combined with SVM, DT, and ANN algorithms, the proposed work achieves good identification performance with an accuracy of 93.75% for all leaf diseases. However, these traditional methods based on simple shallow structures need to rely heavily on manual feature design engineering to obtain satisfactory identification performance. Moreover, these methods lack good performance in the face of large-scale data and complex environment applications. Therefore, more and more researchers have begun to turn their attention to new technologies such as popular deep learning.

### 2.2. Coarse-Grained Deep Learning Recognition Approaches

Deep learning neural networks with multilayer structure and comprehensive optimization strategies have achieved better performance surpassing human recognition or traditional machine learning methods on different visual research aspects. At present, several deep learning-based convolutional neural networks (CNNs), such as AlexNet [26], VGG [32], ResNet [33], and GoogLeNet [34], have been applied in the image recognition of pest and disease species with considerable performance. For example, Mohanty et al. [35] employed the AlexNet and GoogLeNet models to identify various pest categories on the PlantVillage dataset, which obtained a better accuracy rate than other machine learning methods with shallow structures. Chen et al. [36] added a new batch normalization to the VGG network and replaced the fully connected layer with a global pooling layer, improving identification accuracy. Similarly, Yan et al. [37] replaced all fully connected layers of ResNet with global average pooling layers to identify apple leaf disease and pest categories. The experimental results show that the optimization algorithm improves the whole recognition rate of diseases and pests and dramatically shortens the training and recognition time of the model.

In order to improve the generalization of deep learning algorithms in practical applications, many researchers attempted to combine various machine learning strategies to optimize original CNN models with fixed network architecture. For example, Hu et al. [38] firstly introduced the SVM to design an improved conditional deep convolutional generative adversarial network (C-DCGAN) for local area segmentation. Taking lesion images as input, the proposed method could generate new training samples automatically to train the VGG16 model for identifying tea pests and diseases, which achieves better accuracy while relying on only a little image data. From similar thinking, Argüeso et al. [39] studied the identification problem of leaf diseases and pests to handle a small amount of data. Based on the Siamese network with a few-shot learning approach, the proposed method reduced dependence on data quantity while achieving relatively good recognition results.

Moreover, some studies have attempted to apply some knowledge distillation and structural lightweight methods to reduce recognition model parameters, in order to accommodate the real-time application requirements in natural practices. Take Liu et al. [40] for instance; MobileNet and Inception-V3 models with light-weighted structures are migrated simultaneously to construct the deep learning model for plant pest identification. Experimental results show that the optimal method can be transplanted to the Android system on mobile phones, effectively balancing the model's parameters and accuracy. Similarly, Zhang et al. [41] proposed a global pool dilated convolutional neural network (GPDCNN) for plant pest identification. The deep learning network replaces the global convolution of AlexNet with dilated convolution, aiming to reduce the computational complexity and parameter quantity, which are demonstrated to obtain enough recognition performance on cucumber leaf disease and insect images. Although existing coarse-grained methods have achieved some applications in the meta-level identification of plant pests and diseases, they lack the adequate perception for fine-grained features in the absence of particular designs or modules. It is still challenging to implement plant pest and disease recognition in natural settings meeting practical requirements.

### 2.3. Fine-Grained Visual Recognition Methods

Unlike coarse-grained image recognition, fine-grained visual recognition aims to correctly distinguish different sublevel samples within a sizeable meta-level class, which is a significant difficulty in pattern recognition. The critical link of fine-grained identification is discovering some powerful features or local regions, which could represent the potential rules for effectively distinguishing different subclasses with similar appearance and tolerating sample differences of the same class. Fine-grained identification methods are mainly divided into two categories depending on the local labeling approach. The first category is the strongly supervised learning method, which adds extra labels such as label boxes to the image, improving fine-grained identification ability. However, adding extra labels to massive images is a very labor-intensive and time-consuming process, which is not suitable for large-scale datasets. The second kind is the weak supervision method [9], which only relies on the attention method without extra labels to adaptively locate the discriminating part of the target. Such methods have attracted the attention of many researchers. For example, Cruz et al. [20] reduced the number of network layers by modifying convolution blocks as attention modules to contrast the proposed path aggregation network (PANet). This method obtains fine-grained positioning information on different receptive fields to enhance the multiscale features, which is conducive to detecting apple pests and diseases in complex scenes. Similarly, Hughes and Salathé [42] proposed a feature compensation model based on residual neural network (MDFC-ResNet), which grabs multidimensional local information from different granularities into fusing recognition results, achieving a better recognition performance than contrastive deep learning models.

In the above fine-grained methods, the discrimination parts of targets are often used independently. Considering that plant pests and diseases have obvious physiological spatial situations, learning the relationship between different discrimination characteristics can further enhance the discrimination ability. The relationship representation of different local parts can be regarded as a graph structure. Thus, graph neural network plays a vital role in applying non-Euclidean data to analyse the interpretable characteristics, which has excellent research significance in defining the intrinsic relevance of deep learning network nodes and parameters. Recently, deep learning-based graphic networks have developed several model variants, such as graph convolution neural network [43], gated graph neural network [44], graph attention network [45], and graph spatiotemporal network. In the aspect of fine-grained image recognition, Wang et al. [46] proposed the GCL model to exploit and utilize the semantic correlation between regional features fully, designed a cross graph to spread from the network to learn the correlation semantics between different regions, and then enhanced each region by cross-weighted aggregation of other regions. Zhao et al. [25] proposed an effective graph-based relationship discovery method to establish context understanding of high-order relationships. This method regularizes the high-dimensional feature library using high-order constraints of semantic awareness. It embeds the high-order tensor library into the low-dimensional space, which can grasp the context details of fine-grained objects.

In summary, due to the challenging fine-grained problems, traditional machine learning and coarse-grained deep learning methods are unsuitable for plant pest and disease recognition in practical applications. Inspired by graphic neural networks, this paper proposes a graph-related high-order approach via feature aggregation enhancement for fine-grained plant disease and pest identification, aiming to solve the existing identification problems of low accuracy and efficiency. For detailed method descriptions, refer to the following sections.

## 3. Materials and Methods

**Figure 1:**
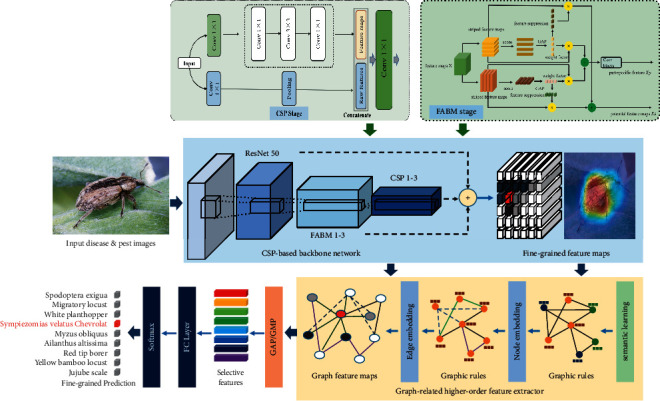
Schematic diagram of the proposed model structure.

This section will introduce the details of the graph-based high-order model constructed for fine-grained plant disease and pest identification. Our proposed GHA-Net consists of three main parts: (1) improved backbone network based on the CSP-stage operations is subjected to extract rich coarse-grained feature maps; (2) feature aggregation enhancement module is proposed to learn more slight features of different parts; and (3) graph-related feature extractor is designed to explore intrinsic relationship among various local features and further mine hidden higher-order features of graph nodes and edges. The overall architecture is shown in Figure 1.

### 3.1. CSP-Based Backbone Network

According to actual application requirements, we apply some data enhancement operations, such as rotate, flip, random crop, Gaussian noise adding, and HSV changing, to enlarge existing image samples before model training. These preprocessing operations can ensure that the deep learning models have sufficient data volume to achieve good generalization and noise resistance to internal and external factor changes. Subsequently, all images are subjected to an improved CSP-based backbone network with the superiority of residual structure. After so many years of theoretical and application demonstrations, a basic knowledge has been recognized by everyone, that is, as the level of the deep neural network is deeper, the model can obtain more scales of receptive fields and rich features, leading to better identification performance. As network structures become more complex and deeper, there is a rapidly growing demand for more training data to fit the explosion of model parameters. However, too many model parameters will not only waste a lot of computing resources but also have little effect on improving model accuracy; due to this, the single path of information propagation in the complex network structure is easy to cause gradient vanishing or explosion.

**Figure 2:**
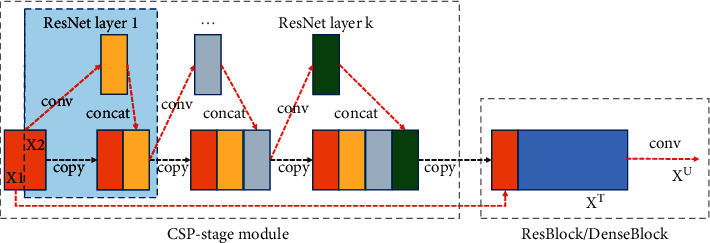
Illustrations of CSP-stage module.

Thus, the CSPNet50 [47], a new variant network of ResNet family, is selected as the backbone network in this paper. The backbone network consists of three CSP stages. Each stage adds several cross-channel branches on the primary residual block, so that a portion of features can directly skip all computational processes, which ensures network performance while reducing the number of parameters. As shown in Figure 2, the CSP-stage module firstly divides the input flow into two parallel parts, X1 and X2. The X2 branch is convolved as the feature of normal convolution to pass through the residual operation. Then, the primary branch X1 is connected to the spanning branch X2 by a 1 × 1 convolution layer. We replace the 1 × 1 convolution of the original model as a channel attention operation to realize the feature interaction, which further enhances the feature extraction ability of the proposed network. After passing through several ResNet layer structures, X1 is aggregated to enhance the learning capability as well as prevent excessive repetitive gradient information, which effectively reduces computational and resource costs. Each CSP-stage module could be combined with any CNN network structure, such as ResBlock or DenseBlock.

### 3.2. Feature Aggregation Enhancement Module

**Figure 3:**
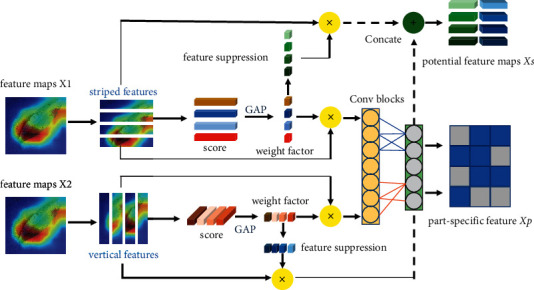
FABM attention module.

To further improve the backbone network's performance and to apply it to fine-grained image classification tasks, we also propose a novel feature aggregation enhancement module on basis of the attention mechanism, which makes the network more focused on the parts with discrimination information in the image. Usually, attention methods can be divided into two categories: strong attention methods and weak attention methods. The strong attention method relies on extra annotation boxes or other annotations, which requires much manual work. The weak attention method is more inclined to automatically add submodules to the network to locate the target position automatically. Our approach focuses on the weak attention method, which only uses the image-level label without adding additional annotations. However, the process of weak attention tends to pay attention to the most significant part, so other inconspicuous but distinguishable parts will be ignored. However, when the essential part is shielded or suppressed, the network will be forced to explore other potential parts. Inspired by Song and Yang [24], we designed a novel structure of weak-supervised attention named as the feature aggregation enhancement module (FABM), which includes an enhanced feature extraction module (EFEM) and feature fusion. In the FABM module, the EFEM module highlights the most prominent part of feature mapping in the current stage to obtain part-specific representations, suppress the representations, and force other potential features to be excavated in the next step. The unique fusion part fuses different elements learned in multiple stages and then obtains feature maps with numerous prominent features. The detailed illustration of FABM module is shown in Figure 3.

Consider feature maps *X* ∈ *R*^*C*×*W*×*H*^ from a specific layer, where C, W, and H denote the number of channels, width, and height, respectively. In the next step, *X* is evenly split into *k* parts along the width and height dimensions, and the parts along the width and height are the same except for the division direction. Take the width division as an example: each striped part is denoted as *X*_*i*_ ∈ *R*^*C*×(*W*/*k*)×*H*^,  *i* ∈ [1, *k*]. Then, we use a 1 × 1 convolution *φ* to explore the importance of each part:

(1) $$\begin{array}{c} A_{\left(i\right)}=\text{Relu}\left(\Phi \left(X_{\left(i\right)}\right)\right)\in R^{1\times \left(W/k\right)\times C}. \end{array}$$

The non-linear function Relu is applied to remove the negative activation. Φ is shared in different stripes, which plays the role of a grader. Then, we take the average of *A*_(*i*)_ as an important factor *b*_0_ of *X*_(*i*)_, i.e.,

(2) $$\begin{array}{c} b_{i}^{\prime }=\text{GAP}\left(A_{\left(i\right)}\right)\in R, \end{array}$$

where GMP denotes global maximum pooling. We use softmax to normalize *B*′=(*b*_1_′,…,*b*_*k*_′)^*T*^:

(3) $$\begin{array}{c} b_{i}=\frac{\exp\left(b_{j}^{\prime }\right)}{\sum_{j=1}^{k}\exp\left(b_{j}^{\prime }\right)}. \end{array}$$

With the normalized importance factor *B*′=(*b*_1_′,…,*b*_*k*_′)^*T*^, the most significant part can be immediately determined. Then, the most significant part of the enhancement feature enhancement is obtained:

(4) $$\begin{array}{c} X_{b}=X+\alpha \left(B⊗X\right), \end{array}$$

where *α*  is a hyperparameter, which controls the degree of enhancement, and ⊗ denotes the multiplication calculated by various elements. Then, a 1 × 1 convolution layer *h* is applied to *X*_*b*_ to get a specific partial representation *X*_*p*_:

(5) $$\begin{array}{c} X_{p}=h\left(X_{b}\right). \end{array}$$

By multiplying the parts with the most stripes, the suppression features *X*_*s*_ are obtained:

(6) $$\begin{array}{c} X_{s}=S⊗X,\\ s_{i}=\left\{\begin{array}{cc} 1-\beta , & \text{if }b_{i}=\text{MAX}\left(B\right),\\ 1, & \text{otherwise,} \end{array}\right. \end{array}$$

where *S*=(*s*_1_, *s*_2_,…, *s*_*k*_) and *β* is a hyperparameter, which controls the degree of suppressing.

The data information carried by multilevel features is very high-dimensional. If each multilevel feature is calculated, it will lead to a large amount of calculation. Therefore, to reduce the amount of calculation, we fuse the multilevel feature information by means of feature addition, to obtain a feature vector with multiple discriminant information:

(7) $$\begin{array}{c} F=\theta \left(\sum_{i=1}^{3}X_{i}\right)\in {\mathbb{R}}^{W_{1}\times H_{1}\times C_{1}}, \end{array}$$

where *F* denotes the feature obtained after fusion, *X*_*i*_ denotes the *i*-th extracted enhancement feature, and *θ* is 1 × 1 convolution.

After multistage fusion, the features obtained already aggregate multiple discriminant parts but ignore the relationship between different semantic channels. In order to increase the interaction between channels, we use the following methods to establish the relationship matrix between channels.

(8) $$\begin{array}{c} M=B\left(\frac{1}{WH}\sum_{i=1}^{WH}F^{T}\varphi \left(F_{i}\right)\right)\in R^{C_{1}\times C_{2}}, \end{array}$$

where *B* denotes normalization, *ϕ* denotes convolution, and *C*_2_ represents the number of channels after convolution.

### 3.3. Graph-Related Higher-Order Feature Extractor

From the FABM module, we can get the feature map with multiple discrimination parts, which can be extracted and processed by a downsampling operation. However, the feature map is still a matrix with high latitude. The high latitude matrix embedded into the low margin using the full connection layer will lead to training many learnable parameters. At the same time, by consulting relevant agricultural pest experts, we know that different pests and diseases have certain hidden information in space, and various pests and diseases have their spatial distribution and shape laws. Based on this, we think that the isolated use of discriminant features cannot learn this information. Inspired by Zhao et al. [25], we used a semantic learning module (SRL) which can further mine higher-order feature maps from raw images. Firstly, the module filters the massive information according to the previous network's feature map to obtain the critical discriminant features representing local parts. Then, we introduce the graph-related convolution network to learn the higher-order semantics between different discrimination parts. The nodes in the graph do not exist in isolation in graph data, so we can build a relationship between different sampling points through a graph convolution network. We construct a graphic dataset using sampling nodes as points and the correlation between points as an adjacency matrix. The higher-order features between parts are implicitly learned and discriminated by spreading on graph convolution network.

Inspired by Zhao et al. [25], we designed a sampler to sample the discrimination parts, which can extract the discrimination parts in the feature map. After obtaining the residual feature map, which aggregates the related features with the original input features, it is sent to the discrimination response layer. Specifically, we introduce a 1 × 1 × *C*_1_ convolution layer and a sigmoid function *σ* to learn discriminative probability maps *S* ∈ *ℝ*^*N*×*H*×*W*^, which express the impact of discriminative regions on the final classification. *C*_1_ is the number of channels in the feature map. Then, each selected patch will be assigned a corresponding discrimination probability value *P*_*ijk*_. The formulation is as follows:

(9) $$\begin{array}{c} P_{ijk}=\left[t_{x},t_{y},t_{k},s_{ijk}\right], \end{array}$$

where [*t*_*x*_, *t*_*y*,_*t*_*k*_] represent the coordinates of each patch and *s*_*ijk*_ denotes the discrimination probability value of the *i*^*th*^ row, the *j*^*th*^ column, and the *k*^*th*^ channel.

**Figure 4:**
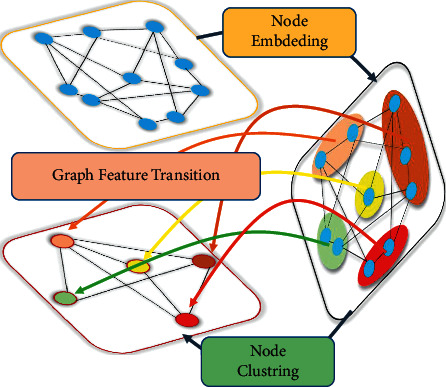
Schematic diagram of graph feature description.

After sampling discriminant feature, a discriminant feature library *𝒦* ∈ *ℝ*^*C*_1×_*C*_2_^ can be constructed. Furthermore, the discriminant feature library *𝒦*={*f*_1_, *f*_2_,…, *f*_*C*_} can be described as a graph with *C*_2_ nodes of *C*_1_ channels as shown in Figure 4. These nodes share many information in essence, so we use graph convolution network to aggregate these features [43]. The relationship between pairs of adjacencies can be defined as

(10) $$\begin{array}{c} A_{i,j}=\frac{\tau {\left(f_{i}\right)}^{T}\cdot \tau \left(f_{j}\right)}{\left\|\tau \left(f_{i}\right)\right\|\left\|\tau \left(f_{j}\right)\right\|}, \end{array}$$

where *τ* denotes the 1 × 1 convolution for dimension transformation, the last adjacency matrix is defined by self-circulation $\tilde{A}=A+I$, and I ∈ ℝ^*C*_1_×*C*_2_^ is an identity matrix. Through this similarity aggregation, the update mode of each node is as follows:

(11) H=ReLuD−12A˜D−12KWg,

where *W*^*g*^ ∈ *ℝ*^*C*_2_×*d*_*h*_^ represent the learnable graph weights with a dimension *d*_*h*_ and $D=\sum_{j}{\tilde{A}}_{i,j}$ is the diagonal matrix for normalization. K denotes the matrix of *𝒦*.

This operation can update the information of each node. At the same time, another purpose of graph embedding is to learn the context understanding of different discrimination parts. We use the following methods to form multiple groups to learn the relationship between contexts:

(12) $$\begin{array}{c} H=\text{ReLu}\left(D^{-1/2}\tilde{A}D^{-1/2}KW^{e}\right), \end{array}$$

where W^*e*^ ∈ ℝ^*d*_*h*_×*C*_*r*_^ represent the learnable graph weights. *G* ∈ ℝ^*C*_1_×*C*_*r*_^ indicates the mapping function of each node from the original space to form a new graph node:

(13) $$\begin{array}{c} Z=H^{T}\frac{e^{G_{i,j}}}{\sum_{j=1}^{C_{r}}\in R^{d_{h}\times C_{r}}},\\ Z=H^{T}\frac{e^{G_{i,j}}}{\sum_{j=1}^{C_{r}}\in R^{d_{h}\times C_{r}}}, \end{array}$$

where *C*_*r*_ represents the number of new embedded graph nodes. We design the softmax layer with the group dimension to accomplish the *C*_*r*_ by the calculation formula *C*_1_/*r*.

This method can efficiently allocate a high-order feature library in low-dimensional manifold and map channel dimension and node dimension at the same time. Then, the remaining connections are grouped and characterized to construct the final embedding $\tilde{Z}=Z+H$. Finally, these embeddings can be finally predicted by applying a GMP or GAP and a classifier.

An obvious problem of infinite granularity classification is overfitting under challenging cases [25]. A preferable class clustering should be based on representative sample sets, while ignoring remote hard samples. This means that we use the centre of multiple samples of a specific class as the average feature to update the network parameters, so the loss function can be defined as follows:

(14) $$\begin{array}{c} L_{\text{loss}}=\frac{1}{NK}\sum_{n=1}^{N}\sum_{k=1}^{K}L_{CE}\left(\left(\frac{y_{n}}{y_{n}}\right)\right)y_{n}, \end{array}$$

where *y*_*n*_ denotes the sample labels, N denotes the number of class, *K* denotes the samples in each class, and L_CE_ represents the cross-entropy function. The representative samples are applied to update the complex parameter features of the whole network, which help improve the model's identification performance with better generalization.

## 4. Experimental Results and Analysis

### 4.1. Experiment Setup

We use three datasets publicly available on the web for experiments: AI Challenger agricultural pest and disease dataset, Cassava leaf disease dataset (Cassava leaves), and IP102 dataset.

#### 4.1.1. AI Challenger Agricultural Disease Dataset

This data contains 27 disease categories on 10 kinds of plant hosts, including apple, cherry, grape, citrus, peach, strawberry, tomato, pepper, corn, and potato. The entire dataset has a total of 47637 image samples. However, the data distribution is highly unbalanced, and the number of pictures in some categories is tiny. Therefore, we cleaned the dataset, eliminated the types with a few images in the dataset, and finally got 31,027 training sets and 4,739 test sets.

#### 4.1.2. Cassava Leaves

This dataset consists of leaf images of cassava plants and contains 21397 cassava leaves. The disease types include cassava brown streak disease (CBSD), cassava mosaic disease (CMD), cassava bacterial blight (CBB), and cassava green mite (CGM). In this paper, the annotated image data are re-divided, including 17,115 images in the training set and 4,282 images in the test set.

#### 4.1.3. IP102

This dataset has a total of 75,222 images, covering 102 different types of pests, including *corn borer*, *red spider mites, whitefly,* and *Spodoptera litura.* At the same time, the crops in the dataset images also contain a variety of crops, including rice, corn, grapes, citrus fruit trees, and mango trees. All images in the classification task are divided into the training set, validation set, and test set. The detection task uses 18,983 annotated images containing bounding boxes, including 15,178 photographs in the training set and 3,798 images in the test set. Since the dataset includes many image data crawled from the network, some of these image data contain some situations such as text occlusion and watermark. These factors will interfere with the training effect of the network, so we culled some parts and finally obtained the training.

In our experiments, the model we used uses ImageNet for pretraining. The images are processed before entering the network, including random cropping, random flipping, and brightness enhancement, to enhance the anti-interference ability of the model. In addition, all images are also normalized, and the image size is set to 448 × 448. In the network training process, the number of iterations is set to 150, and a checkpoint is set every 10 times. The optimizer is SGD, using momentum processing, the weight decay is set to 0.001, and the initial learning rate is set to 0.001. Our experiments are trained end-to-end on 4 NVIDIA P40 GPUs without adding additional part or bounding box annotations, only using image-level labels. In the network structure design, we use the CSPNet50 structure as the backbone network and use the three-stage FABM module in the network structure to extract the features of three different stages for aggregation enhancement.

### 4.2. Evaluation Indicators

In order to objectively evaluate the prediction results of different algorithms, four performance indicators of accuracy, precision, recall, and F1 score are selected to compare the prediction results of each model. ACC is the ratio of the number of correctly labeled items to the total number of observations. PRE is the number of true positives divided by the total number of items belonging to that category. Meanwhile, REC is defined as the number of true positives divided by the total number of items belonging to the category, also known as sensitivity. Through the above three indicators, the number of results in the confusion matrix can be converted into a ratio between 0 and 1, which can be calculated based on true positive (TP), true negative (TN), false positive (FP), and false negative (FN). In addition, another high-level indicator, F1 score, is adopted in this paper, which is the harmonic mean of ACC and REC with a better comprehensive evaluation of multiclass recognition problems. Here are the formula definitions for each indicator:

(15) $$\begin{array}{c} \text{ACC}=\frac{\text{TP}+\text{TN}}{\text{TP}+\text{TN}+\text{FP}+\text{FN}},\\ \text{PRE}=\frac{\text{TP}}{\text{TP}+\text{FP}},\\ \text{REC}=\frac{\text{TP}}{\text{TP}+\text{FN}},\\ F_{1}=2\times \frac{\text{PRC}\times \text{REC}}{\text{PRE}+\text{REC}}. \end{array}$$

The proposed model first selects several regions with the most robust feature performance according to the FABM module. Then, the SRL module tries to extract the discriminative features to construct the correlation between different discriminative parts. Finally, the designed GCN implicitly learns the high-order feature vector to achieve better fine-grained recognition performance. Therefore, to comprehensively evaluate the improvement effect of each module on the proposed method, this paper selects various indicators to analyze the experimental results of our model.

### 4.3. Comparative Results

To verify that the proposed method can identify crop pest species, we set up some experiments to compare the performance of deep stacking networks and other methods. In this section, some coarse-grained image recognition network methods are selected, including VGG [32], Inception [34], ResNet [33], DenseNet [48], CSPNet50 [49], and SeNet [50]; at the same time, several other fine-grained image recognition networks are selected for comparison, including LIR [51], WSAP [52], and FBSM [24]; these networks are trained separately on the three datasets using the same strategy.

The experimental results of AI Challenger are shown in Table 1. The experimental results of the model proposed in this paper are better than those of the commonly used coarse-grained recognition networks, indicating that our network can extract more features than coarse-grained networks. Our network considers learning the discriminative part features of multiple targets as much as possible and suppresses useless features. Therefore, our model is better than other models by comparing coarse-grained networks and some fine-grained methods. In AI Challenger, the best results have been achieved on the dataset, with an accuracy rate of 96.4%, a precision rate of 88.3, a recall rate of 86.7, and an F1 score of 0.87, all of which are optimal.

The experimental results on the Cassava dataset are shown in Table 2. Our experimental results are better than those of other models, except slightly lower than FBSM and LIR. We think this is because the Cassava dataset is more straightforward than the IP102 and AI Challenger. There is one plant category in the Cassava dataset, and the number of pests and diseases is also less. Specifically, the method proposed in this paper focuses more on locating the location of pests and diseases in complex backgrounds. In data with simpler backgrounds, the discriminant parts' enhanced parts receive significant gains. At the same time, since the SRL module learns, the spatial relationship depends on the complexity of the pests. The results show that LIR and FBSM benefit from simpler backgrounds on the Cassava dataset, while in complex environments, such as IP102 and AI Challenger, the method proposed in this paper is more dominant (Table 3).

**Figure 5:**
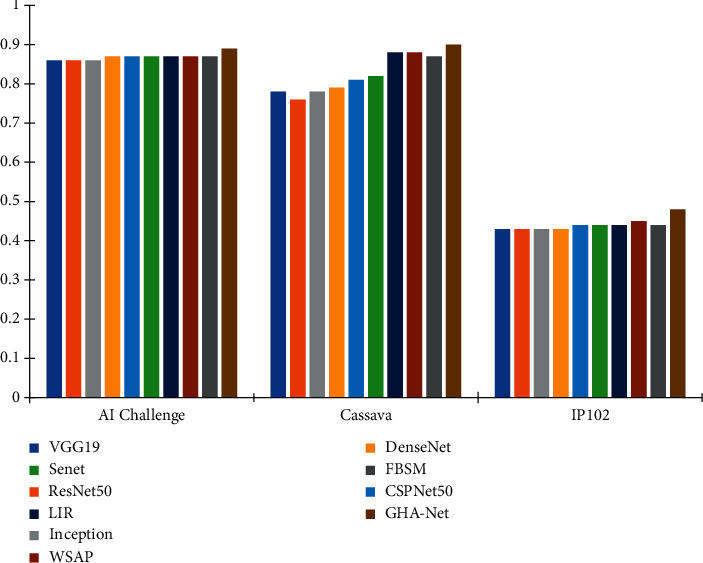
F1 scores of different models on different datasets.

In addition to this, we visually compare the F1 scores of ten models, as shown in Figure 5. It can be seen intuitively that compared with the coarse-grained method, the F1 score of our model is better than that of the coarse-grained method, indicating that our model has better recognition performance and is comparable to other fine-grained methods. In comparison, the F1 score of our model is also comparable to the rest of the fine-grained algorithms, indicating that our model is capable of fine-grained recognition.

**Figure 6:**
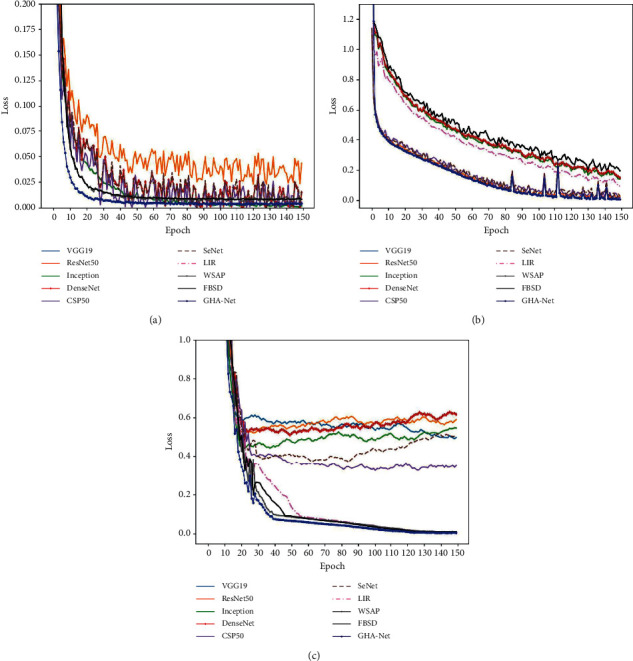
(a) Loss function curves on AI Challenger. (b) Loss function curves on Cassava. (c) Loss function curves on IP102.

As shown in Figure 6, we compared the loss trend of different models on the three datasets. We can see that the loss rate of the proposed model in the early catenary stage is lower than that of other models, since the FABM model and the high-order relation learning module added to the model structure are not pretrained. Hence, the convergence rate in the early stage is relatively slow, but after reaching a certain number of training times, the loss of our model also tends to converge.

**Figure 7:**
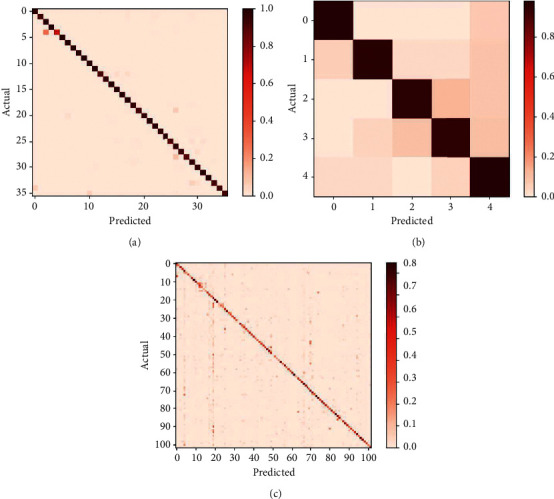
(a) Confusion matrix on AI Challenger. (b) Confusion matrix on Cassava. (c) Confusion matrix on IP102.

To further illustrate the performance of our model, we plotted the confusion matrix of the accuracy rates of the three models, as shown in Figure 7. Our model has a high accuracy rate on each class on the Cassava dataset with the best effect. The worst is the third category, but the accuracy rate has also reached 95%, and the best impact is the 0th category.

**Figure 8:**
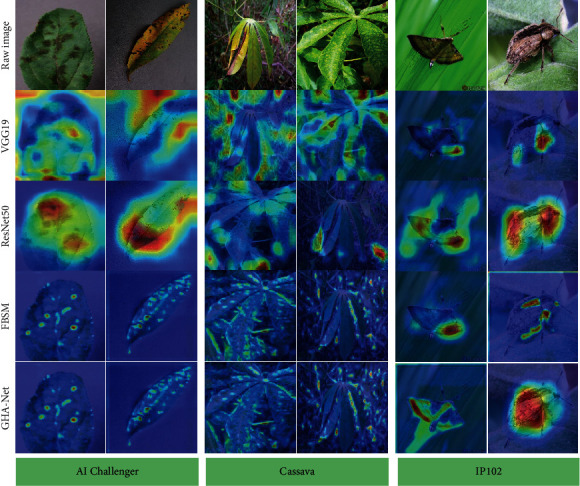
Attention visualization of different models.

To further judge the model's performance proposed in this paper, we visualized part of the model. We considered whether the model played a role through a visual operation. The visualization results are shown in Figure 8. We selected two different samples from each of the three datasets for a total of 6 image samples for visualization. The compared models are VGG19, ResNet50, and FSBM. The heat map shows that the features learned by VGG and ResNet50 contain a lot of background, which is unfavorable for the classification results. At the same time, our model focuses more on the discriminative parts of the target itself, indicating that our model can play the role of learning more discriminative features.

### 4.4. Ablation Analysis

To verify the effectiveness of our model, we conduct ablation experiments on each dataset. The results of our ablation experiments are shown in Table 4, including a feature enhancement module and a spatial higher-order relation learning module. In the absence of fine-grained classification of extra labels or local annotations, we perform feature extraction on the entire image through ResNet50, set it as the baseline network, and then improve the discrimination by eliciting features learned at different residual stages. Finally, the classification accuracy of the network is improved by further extracting and sampling the discriminative features and learning the spatial high-order semantic features between the discriminative parts through the graph. Xia Rong's experiments show that the FABM module can learn more features, and the model accuracy of the FABM module is improved by 1.5%, 5.5%, and 1.7% on the AI Challenger, Cassava, and IP102 datasets, respectively, compared to the baseline network accuracy.

Finally, we introduce the SRL module to learn the implicit relationship between features further, and the network progress is further improved. Ablation experiments prove that the proposed network can understand the discriminative regions, enhance the discriminatory feature values, and improve accuracy. Moreover, we also added a comparison of experimental results using ResNet50 as the backbone network. The experimental results show that after adding the FABM module and the SFL module to ResNet, the experimental results are improved to varying degrees, which shows that our module can be used on other backbone networks, further illustrating the effectiveness of our model.

**Figure 9:**
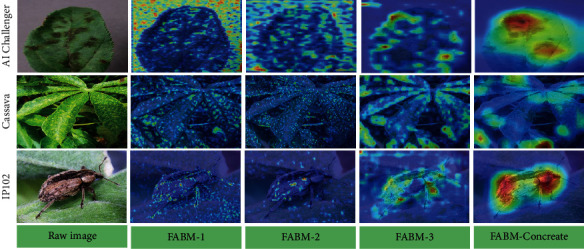
Heat map visualization of different FABM stages.

To further illustrate the effectiveness of our model, we visualize the different stages of the attention module FABM. As shown in Figure 9, our model has three FABM modules, which are denoted as FABM-1, FABM-2, and FABM-3. The aggregation of the three modules is characterized as FABM-Con, as shown in Figure 9. The non-stage FABM can focus on the features of different parts, and the fused FABM can locate multiple discriminative features of the target.

## 5. Conclusion

Aiming to solve the fine-grained identification problem of plant diseases and pests, this research proposes an effective graph-related high-order network with feature aggregation enhancement (GHA-Net) to promote the fine-grained recognition performance of deep learning technology. Based on the improved CSP-stage backbone network, the feature aggregation enhancement module is firstly designed to enhance multilevel attention for learning multiple discriminant features. Meanwhile, the graphic convolution module is constructed to analyse the graph-correlated representation of part-specific interrelationships by regularizing semantic features into the high-order tensor space. Experiments on AI Challenger, Cassava, and IP102 datasets have demonstrated robust and accurate performance in terms of fine-grained plant disease and pest identification. Compared with other deep learning models, the proposed GHA-Net achieves the identification accuracy of 96.4%, 97.4%, and 57.1% on three public datasets, overperforming all compared methods with a minor error, which is verified more suitable for fine-grained identification applications in complex scenes.

The model structure will be optimized to improve the identification performance in further work. The related technologies will be investigated to expand the application scope of the proposed model in intelligent greenhouses and grain warehouses. Also, they can be applied to other fields such as temporal prediction, signal modelling, and control systems [53, 54].

## Figures and Tables

**Table 1:** AI Challenger experiment results of agricultural pests and diseases.

Methods		Accuracy (%)	Precision (%)	Recall (%)	F1
Coarse-grained	VGG19 [32]	94.1	86.2	85.7	0.86
	ResNet50 [33]	94.5	86.4	85.8	0.86
	Inception [34]	95.0	86.9	86.0	0.86
	DenseNet [48]	95.3	86.8	86.3	0.87
	CSPNet50 [49]	95.6	86.9	86.3	0.87
	Senet [50]	95.8	86.9	86.2	0.87
Fine-grained	LIR [51]	95.9	87.0	86.1	0.87
	WSAP [52]	96.2	87.1	86.3	0.87
	FBSD [24]	96.4	87.3	86.4	0.87
	GHA-Net	96.4	88.3	87.4	0.89

**Table 2:** The experimental results of cassava agricultural diseases and pests.

Methods		Accuracy (%)	Precision (%)	Recall (%)	F1
Coarse-grained	VGG19 [32]	88.0	79.0	77.8	0.78
	ResNet50 [33]	89.7	77.0	74.5	0.76
	Inception [34]	88.5	79.3	78.2	0.78
	DenseNet [48]	89.1	79.6	78.4	0.79
	CSPNet50 [49]	92.3	80.1	80.1	0.81
	Senet [50]	94.5	84.3	80.2	0.82
Fine-grained	LIR [51]	98.5	88.3	86.6	0.88
	WSAP [52]	98.4	88.4	87.2	0.88
	FBSD [24]	98.6	87.2	86.5	0.87
	GHA-Net	97.4	89.2	87.9	0.90

**Table 3:** IP102 pest test results.

Methods		Accuracy (%)	Precision (%)	Recall (%)	F1
Coarse-grained	VGG19 [32]	54.1	43.1	42.0	0.43
	ResNet50 [33]	54.7	43.4	42.1	0.43
	Inception [34]	55.3	43.4	42.3	0.43
	DenseNet [48]	55.4	43.6	42.4	0.43
	CSPNet50 [49]	55.6	43.9	42.4	0.44
	Senet [50]	54.3	45.1	42.6	0.44
Fine-grained	LIR [51]	56.9	45.4	42.9	0.44
	WSAP [52]	56.8	45.6	43.3	0.45
	FBSD [24]	56.4	45.4	43.1	0.44
	GHA-Net	57.1	46.7	45.3	0.48

**Table 4:** Ablation experiment results.

Method	ACC (%)		
	AI Challenger	Cassava leaves	IP102
ResNet50	94.5	89.7	54.7
ResNet50+FABM	95.3	94.2	55.9
ResNet50+ SFL	95.0	92.8	55.4
ResNet50+ FABM + SFL	96.0	96.1	56.9
CSP	95.6	92.3	55.6
CSP + FABM	96.0	95.2	56.4
CSP + SFL	95.9	93.6	56.0
CSP + FABM + SFL	96.4	96.4	57.1

## Data Availability

The datasets presented in this study are available from the following links: AI Challenger—https://aistudio baidu com/aistudio/datasetdetail/76075; Cassava—https://www kaggle.com/c/cassava-disease/overview; and IP102—https://github com/xpwu95/IP102.
